# Meta-analysis of the efficacy and safety of Xihuang Pills/capsules in adjuvant treatment of uterine cervical neoplasms

**DOI:** 10.1097/MD.0000000000034846

**Published:** 2023-08-25

**Authors:** Huirong Xu, Guangyu Tian, Chunli Wu, Xiaowen Sun, Kejian Li

**Affiliations:** a College of Traditional Chinese Medicine, Shandong University of Traditional Chinese Medicine, Jinan, P.R. China; b Jinan city Hospital of Integrated Traditional Chinese and Western Medicine, Jinan, P.R. China.

**Keywords:** adverse reaction, effectiveness, meta-analysis, uterine cervical neoplasms, Xihuang Pills/Capsules

## Abstract

**Background:**

Xihuang Pills/Capsules have a longstanding history of utilization in traditional Chinese medicine (TCM) for treating cancer. Nevertheless, a comprehensive investigation is required regarding the specific impacts and safety of Xihuang Pills/Capsules in individuals with uterine cervical neoplasms. Thus, conducting a meta-analysis is essential to evaluate the clinical effectiveness of combining Xihuang Pills/Capsules with Western medicine in patients with cervical neoplasms.

**Methods:**

The research involved searching 5 English and 4 Chinese databases for randomized controlled trials (RCTs) investigating the use of Xihuang Pills/Capsules in conjunction with Western medicine for treating uterine cervical neoplasms. Subsequently, statistical analysis was carried out using Review Manager software (version 5.3).

**Results:**

This research encompassed 10 RCTs involving 937 patients. The findings revealed that the combination of Xihuang Pills/Capsules with Western medicine treatment led to improvements in various aspects of the patients’ condition. Specifically, there was an enhancement in the short-term efficacy rate (risk ratio [RR] = 1.14, 95% confidence interval [CI]: 1.06–1.22, *P* = .0003), Karnofsky performance score (KPS) (mean difference [MD] = 5.90, 95% CI: 0.54–11.26, *P* = .03), survival rates, CD3+, CD3 + CD4+, CD3 + CD8+, CD3–CD56 + cells, and immunoglobulin M in patients with uterine cervical neoplasms. Moreover, the combination treatment resulted in a reduction of adverse reactions, including gastrointestinal reactions (RR = 0.52, 95% CI: 0.42–0.64, *P* < .00001), radiation proctitis (RR = 0.47, 95% CI: 0.33–0.68, *P* < .0001), myelosuppression (RR = 0.41, 95% CI: 0.26–0.64, *P* < .0001), as well as carcinoembryonic antigen (CEA) and squamous cell carcinoma antigen (SCC-Ag) levels. Additionally, the treatment exhibited an inhibitory effect on white blood cells (WBCs) and platelets (PLTs).

**Conclusion:**

The amalgamation of Xihuang Pills/Capsules with conventional anti-tumor therapy proves to be both effective and safe in the treatment of cervical neoplasms. However, further validation through high-quality RCTs is necessary to substantiate these findings.

## 1. Introduction

Uterine cervical neoplasms represent the most prevalent gynecological tumors, mainly attributed to human papillomavirus (HPV) infection, and stand as the fourth most common cancer in women, trailing breast, colorectal, and lung cancers. Despite its preventable nature, cervical neoplasms persist as the second leading cause of death among female cancer patients aged 20 to 39 years.^[1]^ Recent statistics from the International Agency for Research on Cancer indicate that approximately 527,000 women are diagnosed with cervical cancer annually, with around 311,000 succumbing to the disease.^[2]^ This condition is not only detrimental to women’s health but also exhibits a notably low 5-year survival rate. Hence, timely diagnosis and treatment are of paramount importance, as delayed interventions can adversely impact treatment outcomes.^[3]^ Non-surgical regimens have now become the prevailing approach for intermediate and advanced cervical cancer, encompassing radiotherapy, chemotherapy, targeted therapy, and immunotherapy as the most commonly employed modalities.^[4]^ Radiotherapy and chemotherapy have gained clinical recognition as fundamental treatment methods, often administered in tandem.^[5]^ Despite their relatively significant clinical effects, accumulating research suggests that these therapies are associated with numerous complications, toxicities, and side effects,^[6]^ including myelosuppression,^[7–9]^ gastrointestinal reactions,^[10]^ radiation proctitis, and radiation cystitis.^[11]^ However, over time, cervical neoplasms may develop resistance to traditional treatments and certain individuals may prove unresponsive to chemotherapy or radiotherapy.^[12,13]^ These factors limit the effectiveness of these treatments and impact the prognosis of cervical cancer patients. Therefore, a pivotal point for discussion in clinical practice centers on the selection of scientific and effective treatment plans to improve clinical efficacy while minimizing adverse treatment reactions, ultimately enhancing patient prognoses.

Chinese materia medica, an integral component of traditional Chinese medicine (TCM), has been utilized since ancient times to treat various diseases. Within comprehensive cancer treatment in China, Chinese medicinal herbs have shown the ability to enhance the efficacy of Western medical treatment, reduce the toxicity of radiotherapy and chemotherapy, alleviate tumor-induced symptoms and pain, and prolong the survival and quality of life for late-stage cancer patients.^[14,15]^

Xihuang Pill, a traditional TCM anti-cancer prescription and the first proprietary Chinese medicine for anti-cancer, has been employed in clinical practice in China for over 300 years. Its traditional usage was recorded in “Wai Ke Zheng Zhi Quan Sheng Ji” (Collection of Surgical Syndrome and Treatment) and was originally developed by Wang Hongxu during the Qing Dynasty. Comprising 4 Chinese medicinal herbs – Niu Huang (Calculus Bovis), She Xiang (Moschus), Ru Xiang (Olibanum), and Mo Yao (Commiphora Myrrha) – Xihuang Pill not only hinders tumor proliferation and metastasis but also protects non-tumor cells from conventional therapeutic drugs.^[16]^ Presently, Xihuang Pill is available in 2 forms: pills and capsules. In ancient China, Xihuang Pill was predominantly used to treat furunculosis, scrofula, subcutaneous nodules, and cancer. Today, Xihuang Pills/Capsules find extensive application in clinical research as adjuvant therapy for various malignant tumors, including breast cancer, colorectal cancer, ovarian cancer, lung cancer, liver cancer, and lymphoma.^[17–22]^ However, there is currently a lack of evidence-based medical studies on uterine cervical neoplasms. Consequently, to assess the clinical safety of Xihuang Pills/Capsules when combined with other therapies for treating cervical neoplasms, this study conducted a meta-analysis of randomized controlled trials (RCTs). The aim was to explore the safety of Xihuang Pills/Capsules as adjuvant treatment for cervical cancer, as well as its impact on tumor progression, quality of life, immunity, and prognosis.

## 2. Methods

The present meta-analysis adhered to the guidelines outlined in the Preferred Reporting Items for Systematic Reviews and Meta-Analyses (PRISMA) 2020 statement.^[23]^ The research protocol was registered and recorded in the PROSPERO database under the identifier CRD42022368098 (http://www.crd.york.ac.uk/prospero/#myprospero).

### 2.1. Inclusion criteria

The meta-analysis incorporated clinical trials that satisfied the following criteria: patients in each trial were diagnosed with cytologically or pathologically confirmed uterine cervical neoplasms; the experimental group received a combination of Xihuang Pills/capsules and Western medicine treatment, while the control group received Western medicine treatment alone; the trials were RCTs, and the outcomes assessed included immediate tumor response, functional status evaluated using the Karnofsky performance score (KPS), immune system response, reduction in adverse events of chemoradiotherapy such as myelosuppression and gastrointestinal reactions, radiation proctitis, and prognosis.

### 2.2. Exclusion criteria

The meta-analysis excluded trials based on the following criteria: trials that did not fulfill the aforementioned inclusion criteria; patients in the treatment group who received oral TCM other than Xihuang Pills/Capsules and compound interventions; non-original research or duplicate publications; trials with irrelevant outcomes or erroneous data; and basic studies or literature reviews.

### 2.3. Sources of information and search strategy

A comprehensive search was conducted across the following databases: PubMed, Cochrane Library, Embase, MEDLINE, Web of Science, China National Knowledge Infrastructure, Wanfang Database, VIP Database, and China Biology Medicine. The retrieval period encompassed from the establishment of these databases until May 2023, without any language or publication restrictions. An example of the PubMed search strategy is as follows:

#1 Search: ((((((((((((((((((((((((((uterine cervical neoplasms [MeSH Terms]) OR (Cervical Neoplasm, Uterine [Title/Abstract])) OR (Cervical Neoplasms, Uterine [Title/Abstract])) OR (Neoplasm, Uterine Cervical [Title/Abstract])) OR (Neoplasms, Uterine Cervical [Title/Abstract])) OR (Uterine Cervical Neoplasm [Title/Abstract])) OR (Neoplasms, Cervical [Title/Abstract])) OR (Cervical Neoplasms [Title/Abstract])) OR (Cervical Neoplasm [Title/Abstract])) OR (Neoplasm, Cervical [Title/Abstract])) OR (Neoplasms, Cervix [Title/Abstract])) OR (Cervix Neoplasms [Title/Abstract])) OR (Cervix Neoplasm [Title/Abstract])) OR (Neoplasm, Cervix [Title/Abstract])) OR (Cancer of the Uterine Cervix [Title/Abstract])) OR (Cancer of the Cervix [Title/Abstract])) OR (Cervical Cancer [Title/Abstract])) OR (Uterine Cervical Cancer [Title/Abstract])) OR (Cancer, Uterine Cervical [Title/Abstract])) OR (Cancers, Uterine Cervical [Title/Abstract])) OR (Cervical Cancer, Uterine [Title/Abstract])) OR (Cervical Cancers, Uterine [Title/Abstract])) OR (Uterine Cervical Cancers [Title/Abstract])) OR (Cancer of Cervix [Title/Abstract])) OR (Cervix Cancer [Title/Abstract])) OR (Cancer, Cervix [Title/Abstract])) OR (Cancers, Cervix [Title/Abstract])

#2 Search: ((((((((((xihuang [Supplementary Concept]) OR (xi huang [Title/Abstract])) OR (xihuangwan [Title/Abstract])) OR (xihuang pill [Title/Abstract])) OR (xihuang capsule [Title/Abstract])) OR (xi huang pill [Title/Abstract])) OR (xi huang capsule [Title/Abstract])) OR (xihuang pills [Title/Abstract])) OR (xihuang capsules [Title/Abstract])) OR (xi huang pills [Title/Abstract])) OR (xi huang capsules [Title/Abstract])

#3 Search: (((randomized controlled trial [Publication Type]) OR (controlled clinical trial [Publication Type]) OR (randomized [Title/Abstract] OR randomized [Title/Abstract]) OR (placebo [Title/Abstract]) OR (drug therapy [MeSH Subheading]) OR (randomly [Title/Abstract]) OR (trial [Title/Abstract]) OR (groups [Title/Abstract])) NOT (animals [MeSH Terms] NOT humans [MeSH Terms]))

#1 and #2 and #3

### 2.4. Study selection and data extraction

Endnote X9.3 (Thomson Corporation, Stanford, CT) was employed to manage and eliminate duplicate documents identified through the database search. Two independent reviewers, H.X. and G.T., conducted a preliminary review of eligible documents based on their titles and abstracts. Full texts of potentially relevant studies were retrieved and screened against the inclusion criteria. Any disagreements regarding the eligibility of specific studies were resolved through discussion or consultation with a third researcher, C.W.

Studies were selected based on the criteria stipulated in the Cochrane Handbook for Systematic Reviews of Interventions. Two reviewers utilized a standardized data extraction form to extract relevant information. Any discrepancies were resolved through consensus or consultation with another reviewer. The extracted data included the first author and publication date, random sequence generation, sample size, participant age, treatment details and comparisons, treatment duration, outcomes, adverse reactions, and follow-up period.

### 2.5. Study risk of bias assessment

The selection of clinical trials followed criteria outlined in the Cochrane Handbook for Systematic Reviews of Interventions. Two authors independently accessed the full texts of potentially relevant studies and assessed them against the inclusion criteria. Any disagreements were resolved through discussion or consultation with another researcher. The criteria comprised 7 items: selection bias (random sequence generation and allocation concealment), performance bias (blinding of participants and personnel), detection bias (blinding of outcome assessment), attrition bias (incomplete outcome data), reporting bias (selective reporting), and other bias. Each item was assigned a quality rating of low, high, or unclear risk. Discrepancies between the 2 reviewers were resolved through discussion with another reviewer until consensus was achieved.

### 2.6. Statistical analysis

The meta-analysis was performed using Review Manager 5.3 software (Cochrane Collaboration, Copenhagen, Denmark). For dichotomous data, the risk ratio (RR) with a 95% confidence interval (CI) was utilized, while continuous outcomes were analyzed using the mean difference (MD) with a 95% CI. Heterogeneity was assessed using the *I*^2^ test, with a value of *I*^2^ ≥ 50% and *P* < .05 indicating the presence of heterogeneity, in which case a random-effects model was employed. In the absence of heterogeneity (*I*^2^ < 50%, *P* ≥ .05), a fixed-effects model was used for the analysis. Egger’s test was conducted to examine publication bias (generally when more than 10 studies were included).

## 3. Results

### 3.1. Study selection

**Figure 1.**
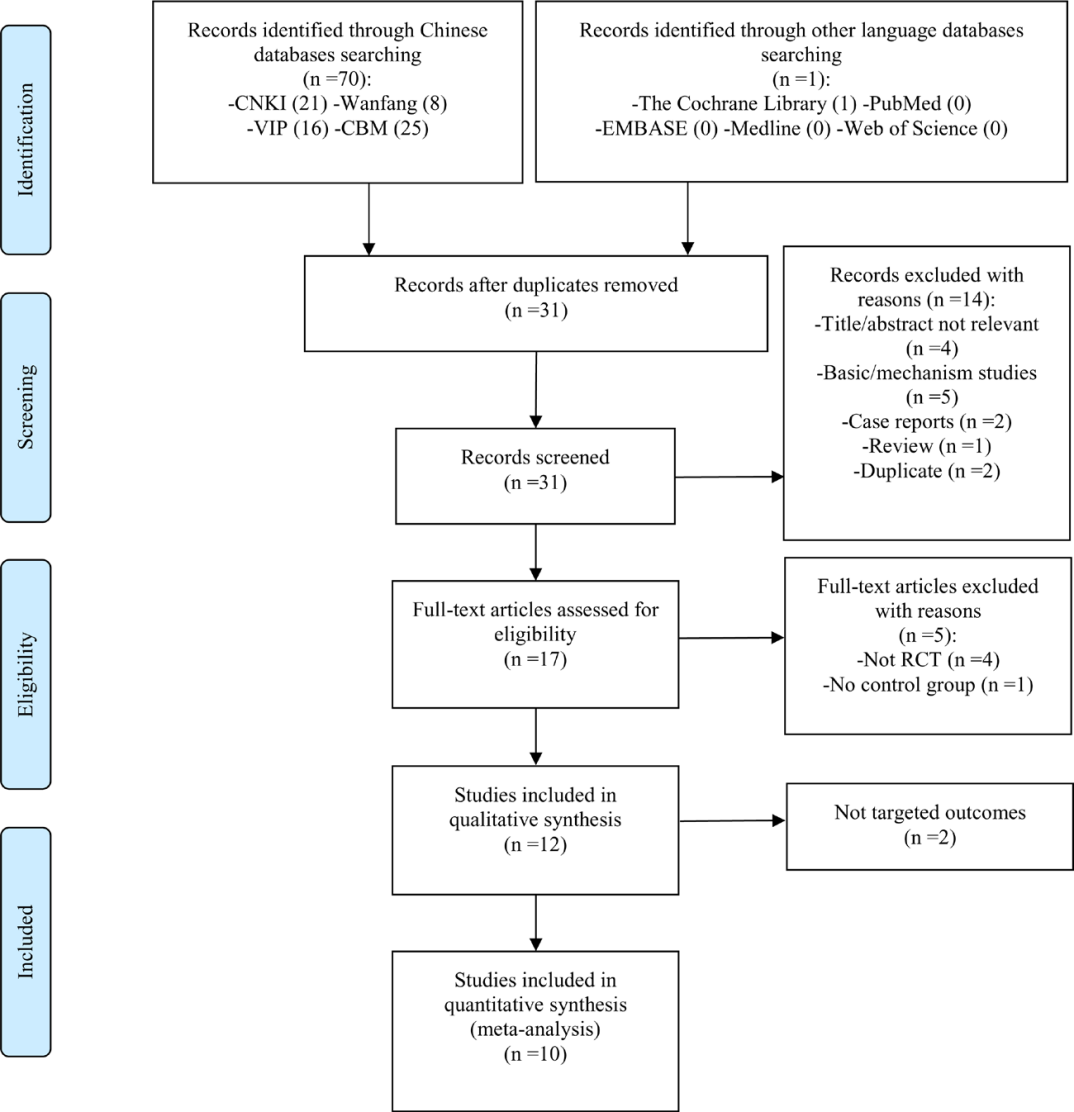
PRISMA flow diagram of included studies. PRISMA = Preferred Reporting Items for Systematic reviews and Meta-Analysis, RCTs = randomized controlled trials.

Through electronic database searches, we acquired 71 relevant documents, consisting of 70 Chinese articles and 1 English article. After eliminating 40 duplicate articles using Endnote X9.3, we evaluated the titles and abstracts of the remaining studies. Among these, 4 studies were deemed irrelevant, 5 were categorized as basic/mechanistic studies, 2 were case reports, 1 was a literature review, and 2 were duplicate publications. Subsequently, the full texts of the remaining 17 studies were examined. From these, 5 articles were excluded due to not being RCTs and one lacked a control group. Further investigation revealed that 2 trials did not achieve the targeted outcomes. Ultimately, 10 RCT studies were considered eligible for qualitative analysis (Fig. 1).^[24–33]^

### 3.2. Study characteristics

The 10 studies were published in the Chinese literature between 2009 and 2022 (Table 1). These trials encompassed a total of 937 patients diagnosed with mid-to late-stage cervical cancer. Among them, 470 patients underwent treatment with Xihuang Pills/Capsules combined with Western medicine (which included radiotherapy and/or chemotherapy, with one study involving radical hysterectomy), while the remaining 467 patients received Western medicine treatment alone.

**Table 1:** Characteristics of 10 included trails.

Study	Sample size (EG/CG)	Control group intervention	Experimental group intervention	Duration (wk)	Assessment of outcomes
Zhang XY, et al, 2022^[24]^	60 (30/30)	RT	XHC + RT	8	Tumor response, CD3 + CD8+, CD3 + CD4+, CD3+, CD3-CD56+, tumor marker (SCC-Ag, CYFRA21–1, CEA), miR-155, miR-24, adverse reaction, 1-year/2-year/3-year survival rate
Tai, et al, 2017^[25]^	42 (21/21)	RT	XHC + RT	4	Symptom curative effect of TCM, tumor response, CD3+, CD3 + CD4+, CD3 + CD8+, CD3－CD56+, IL－6
Peng, et al, 2018^[26]^	186 (93/93)	CT	XHC + CT	8	Recurrence and metastasis rates, adverse reaction, tumor response
Qiu, et al, 2017^[27]^	102 (52/50)	RT	XHC + RT	7	KPS, tumor response, adverse reaction, 1-year/2-year survival rate
Li, et al, 2009^[28]^	89 (45/44)	RT	XHC + RT	8	Tumor response, adverse reaction
Chen J, et al, 2016^[29]^	154 (77/77)	RT + CT	XHP + RT + CT	6	Tumor response, local control rate, adverse reaction, 1-year/2-year survival rate
Zhang GH, 2020^[30]^	78 (39/39)	CT	XHP + CT	12	Tumor response, CD4+, CD8+, CD4+/CD8+, IgA, IgM, tumor marker (CEA, CA199, SCC-Ag), QOL, CFS, adverse reaction
Chen QM, et al, 2016^[31]^	62 (30/32)	RT + CT	XHP + RT + CT	5	Tumor response, QOL, adverse reaction, CD4+, CD8+, CD4 + CD25+, CD4+/CD8
ZhangJ G, et al, 2018^[32]^	84 (43/41)	RT + CT	XHC + RT + CT	9	Incidence rate of radiation proctitis, grade of radiation proctitis
Sang, et al, 2017^[33]^	80 (40/40)	Radical hysterectomy + CT	XHP + radical hysterectomy + CT	6	Tumor response, 3-month and 6-month survival and recurrence rates, CD3+, CD4+, CD8+, IgA, IgG, IgM, KPS, VAS, adverse reaction

CA199 = carbohydrate antigen 199, CEA = carcinoembryonic antigen, CFS = cancerous fatigue score, CG = control group, CT = chemotherapy, CYFRA21-1 = cytokeratin 19 fragment antigen, EG = experimental group, Ig = immunoglobulin, KPS = Karnofsky performance score, QOL = quality of Life, RT = radiotherapy, SCC-Ag = squamous cell carcinoma antigen, TCM = traditional Chinese medicine, VAS = visual analogue scale, XHC = Xihuang Capsules, XHP = Xihuang Pills.

### 3.3. Risk of bias in studies

**Figure 2.**
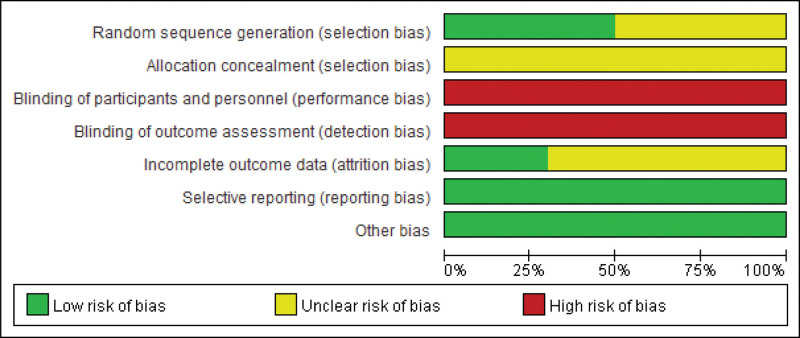
Graph indicating risk of biasness.

**Figure 3.**
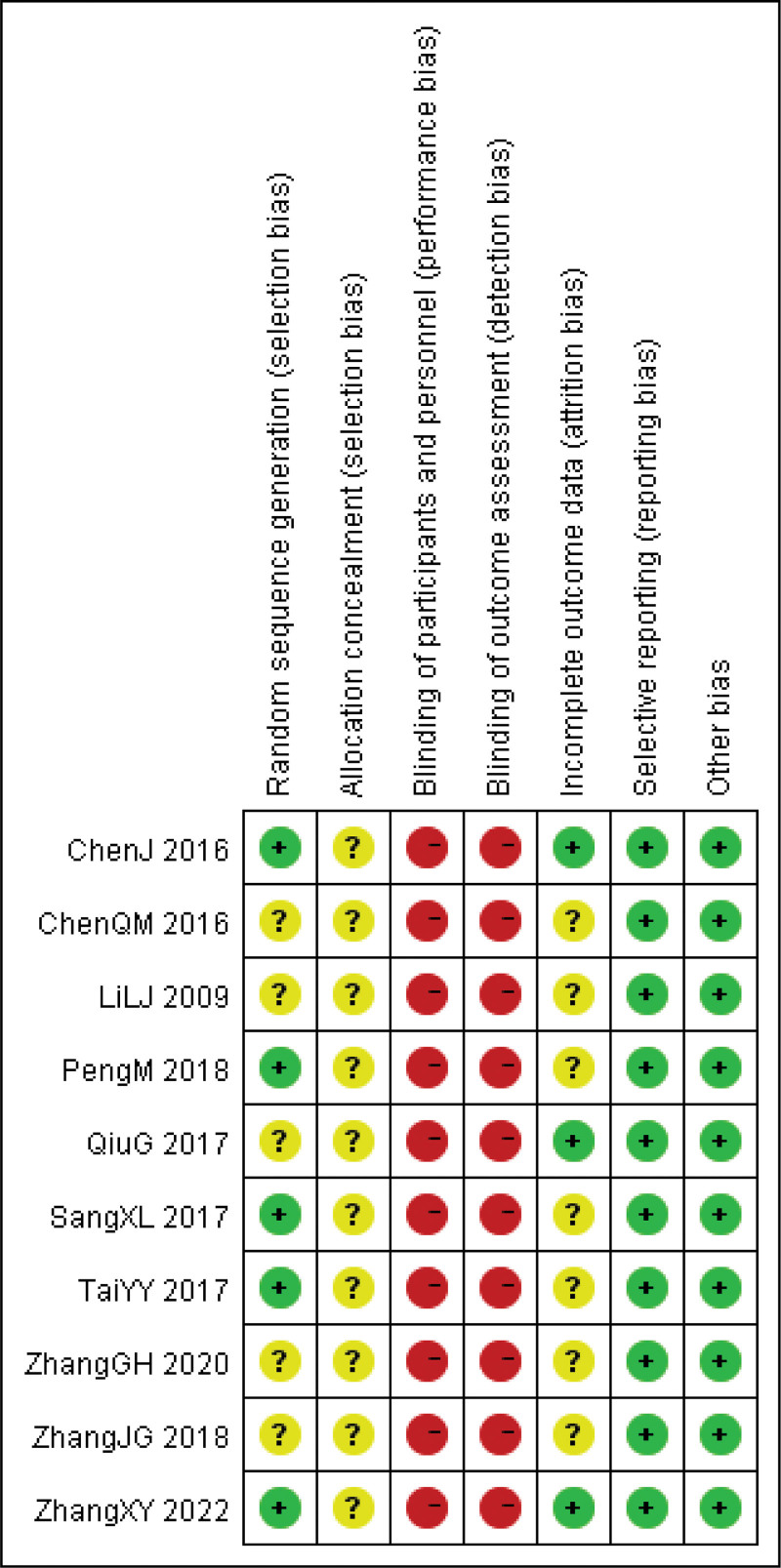
Summary of bias risk.

All the included studies were described as randomized, with 5 trials specifically mentioning the use of random number tables for randomization.^[24–26,29,33]^ The randomization process was carried out, and the patients were considered to be at minimal risk. However, none of the documents provided information on allocation concealment. We made attempts to contact the authors through email and phone but were unable to establish communication. Among the studies, 3 reported on the follow-up process, indicating complete outcome data.^[24,27,29]^ Furthermore, all the studies presented comprehensive details about the measurements used in the methodology, ensuring comparability of baseline data. The evaluation findings are displayed in Figures 2 and 3.

### 3.4. Effects of the intervention

#### 3.4.1. Tumor response.

**Figure 4.**
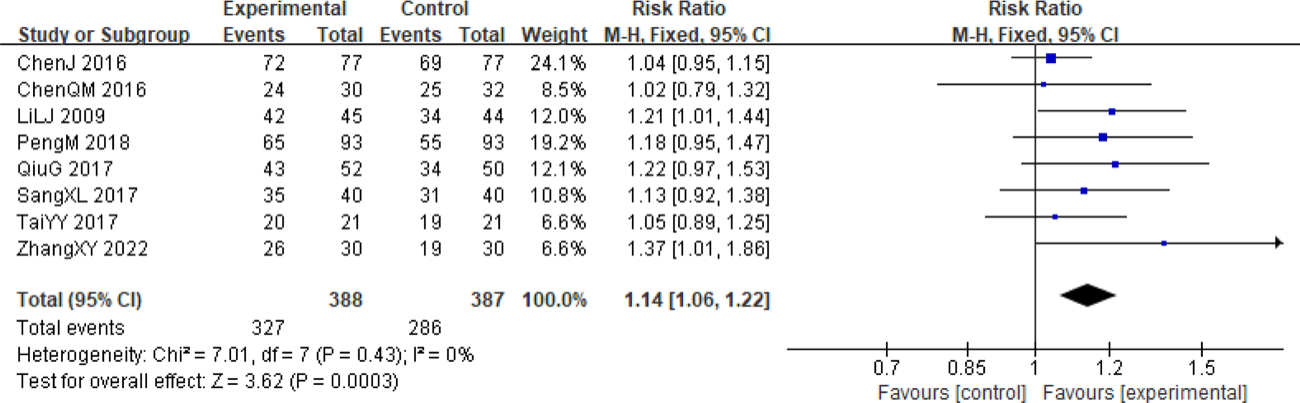
Immediate tumor response (CR + PR) during cervical cancer treatment. CI = confidence interval, CR = complete response, PR = partial responses.

**Figure 5.**
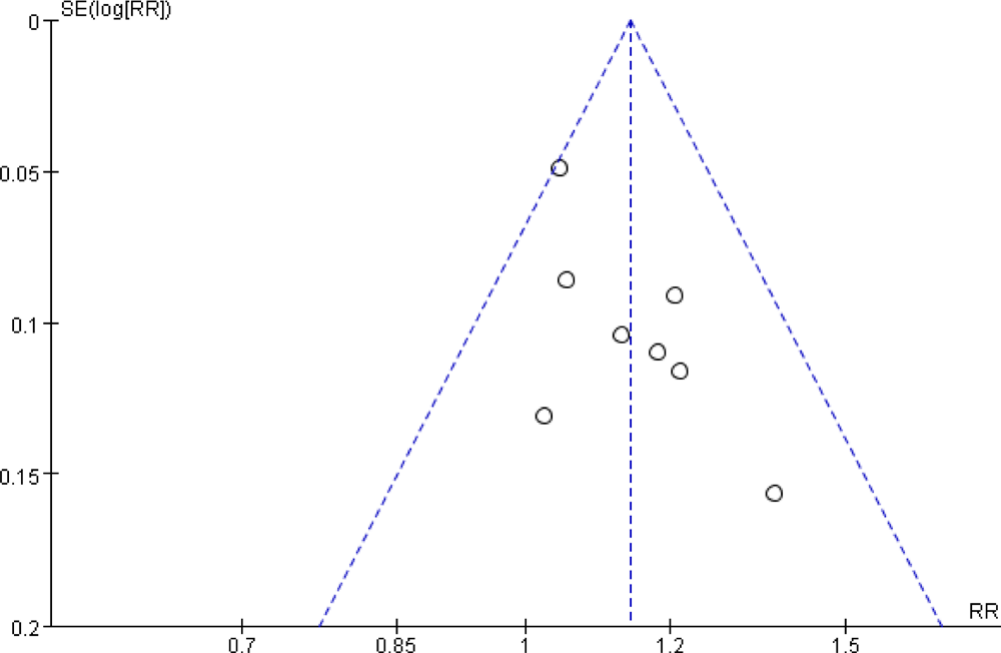
A funnel plot of the immediate tumor response (CR + PR) during cervical cancer treatment. CI = confidence interval, CR = complete response, PR = partial responses.

Eight studies reported on the short-term tumor responses in both groups.^[24–29,31,33]^ Among these, 327 patients who received a combination of Xihuang Pills/Capsules with Western medicine treatment showed a complete response or partial response, whereas 286 patients treated with Western medicine alone achieved complete response or partial response. The combined therapy of Xihuang Pills/Capsules with Western medicine exhibited a significantly higher short-term effective rate in patients with uterine cervical neoplasms compared to the control group (RR = 1.14, 95% CI: 1.06–1.22, *P* = .0003, 775 patients). The studies demonstrated good homogeneity (χ^2^ = 7.01, *P* = .43, *I*^2^ = 0%) (Fig. 4). However, a funnel chart analysis of the 8 articles indicated a potential publication bias, as the graph showed a roughly symmetrical distribution (Fig. 5).

#### 3.4.2. Functional status.

**Figure 6.**
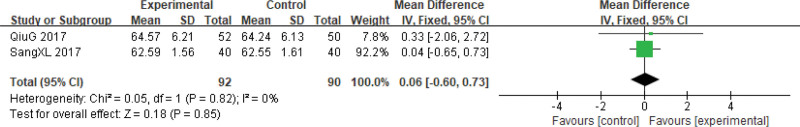
Pretreatment KPS. CI = confidence interval, KPS = Karnofsky performance score.

**Figure 7.**
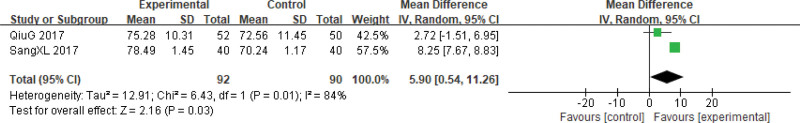
Post-treatment KPS. CI = confidence interval, KPS = Karnofsky performance score.

Two articles reported the pre- and post-treatment KPS data in both groups.^[27,33]^ The pretreatment KPS data showed no significant difference between the 2 groups (MD = 0.06, 95% CI: –0.60 to 0.73, *P* = .85, 182 patients), and the statistical heterogeneity was minimal (χ^2^ = 0.05, *P* = .82, *I*^2^ = 0%) (Fig. 6). However, the experimental group exhibited a significantly higher post-treatment KPS compared to the control group (MD = 5.90, 95% CI: 0.54–11.26, *P* = .03, 182 patients). Heterogeneity testing indicated a χ^2^ of 6.43, *P* value of .01, and *I*^2^ of 84% (Fig. 7). These findings suggest that combining Xihuang Pills/Capsules with Western medicine treatment could lead to improvements in KPS and overall quality of life for patients with cervical neoplasms.

#### 3.4.3. Reduction in adverse events.

**Figure 8.**
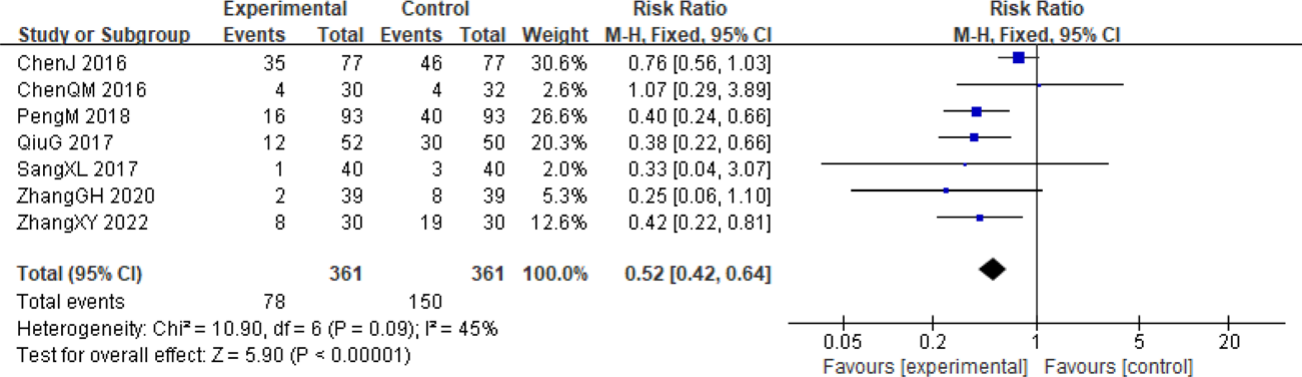
Reactions in the gastrointestinal tract during cervical cancer treatment. CI = confidence interval.

Gastrointestinal reactions are known to be significant adverse effects of chemoradiotherapy.^[34]^ However, in patients treated with Xihuang Pills/Capsules combined with Western medicine, the frequency of digestive tract reactions (such as nausea, vomiting, and diarrhea) was markedly reduced (RR = 0.52, 95% CI: 0.42–0.64, *P* < .00001, 7 studies, 722 patients).^[24,26,27,29–31,33]^ The heterogeneity test showed a value of χ^2^ = 10.90, *P* = .09, and *I*^2^ = 45% (Fig. 8).

**Figure 9.**
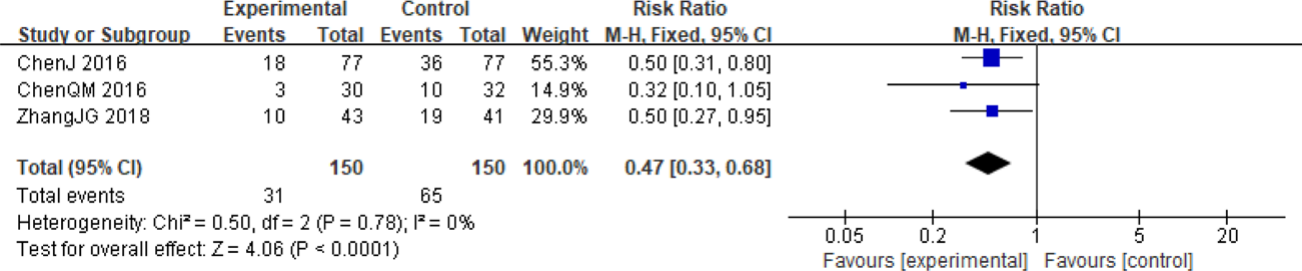
Radiation proctitis during cervical cancer treatment. CI = confidence interval.

Radiation proctitis is a common side effect of chemoradiotherapy in uterine cervical neoplasms.^[35]^ However, when Xihuang Pills/Capsules were combined with chemoradiotherapy, the incidence of radiation proctitis significantly decreased compared to the group receiving chemoradiotherapy alone (RR = 0.47, 95% CI: 0.33–0.68, *P* < .0001, 3 studies, 300 patients).^[29,31,32]^ There was minimal variation among the studies (χ^2^ = 0.50, *P* = .78, *I*^2^ = 0%) (Fig. 9).

**Figure 10.**
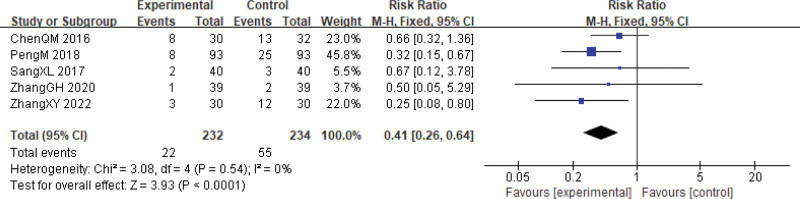
Myelosuppression during cervical cancer treatment. CI = confidence interval.

Myelosuppression is a common side effect in uterine cervical neoplasia.^[36]^ It was observed in 5 studies.^[24,26,30,31,33]^ The combined results revealed a significantly lower incidence of myelosuppression in the experimental group compared to the control group (RR = 0.41, 95% CI: 0.26–0.64, *P* < .0001, 5 studies, 466 patients). Heterogeneity testing showed χ^2^ = 3.08, *P* = .54, and *I*^2^ = 0% (Fig. 10).

**Figure 11.**
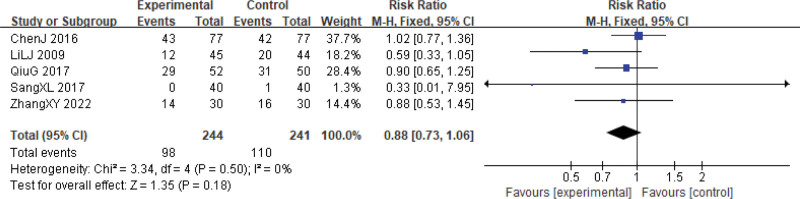
WBCs reductions during cervical cancer treatment (toxicity grades I–II). CI = confidence interval, WBCs = white blood cells.

**Figure 12.**
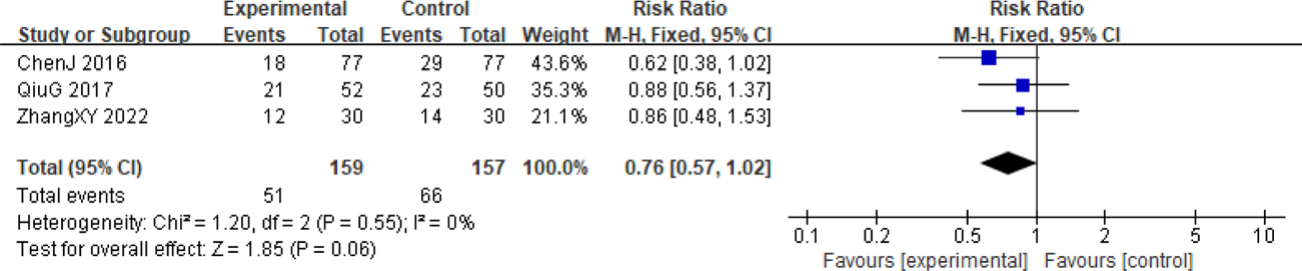
PLTs are reduced during cervical cancer treatment (toxicity grades I–II). CI = confidence interval, PLTs = platelets.

There were 5 studies investigating the reduction of white blood cell (WBC) inhibition and 3 studies on platelet (PLT) inhibition.^[24,27–29,33]^ Our analysis revealed that the reductions in these 2 indicators at grades I–II did not differ significantly between the 2 groups (WBCs, RR = 0.88, 95% CI: 0.73–1.06, *P* = .18, 5 studies, 485 patients; PLTs, RR = 0.76, 95% CI: 0.57–1.02, *P* = .06, 3 studies, 316 patients), and there was no significant heterogeneity among the studies (WBCs, χ^2^ = 3.34, *P* = .50, *I*^2^ = 0%; PLTs, χ^2^ = 1.20, *P* = .55, *I*^2^ = 0%) (Figs. 11 and 12).

**Figure 13.**
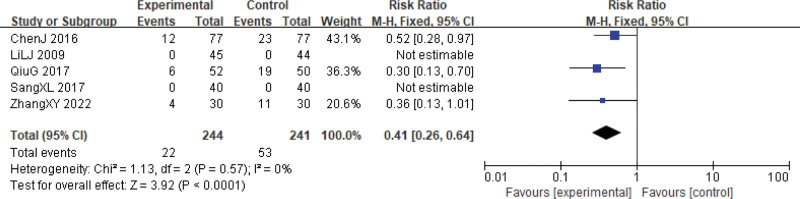
WBCs reductions during cervical cancer treatment (toxicity grades III–IV). CI = confidence interval, WBCs = white blood cells.

**Figure 14.**
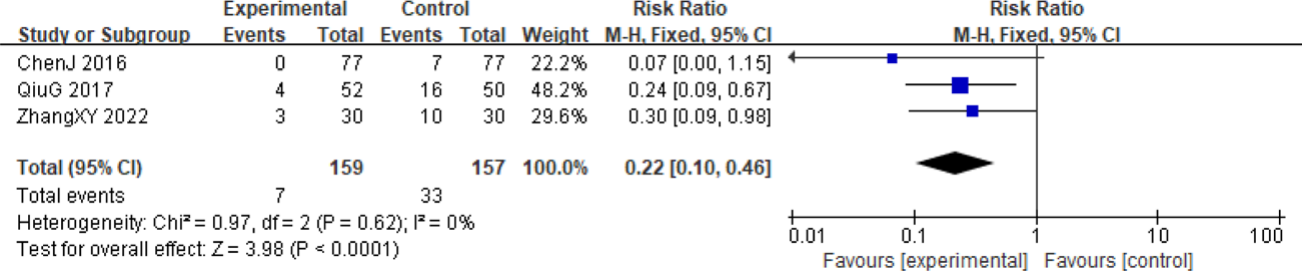
PLTs reduction during cervical cancer treatment (toxicity grades III–IV). CI = confidence interval, PLTs = platelets.

However, for reductions in WBC and PLT inhibitions at grades III to IV, the Xihuang Pills/Capsules combined with Western medicine treatment group showed significantly lower levels (WBCs, RR = 0.41, 95% CI: 0.26–0.64, *P* < .0001, 5 studies, 485 patients; PLTs, RR = 0.22, 95% CI: 0.10–0.46, *P* < .0001, 3 studies, 316 patients). No significant heterogeneity was observed among the studies for these outcomes (WBCs, χ^2^ = 1.13, *P* = .57, *I*^2^ = 0%; PLTs, χ^2^ = 0.97, *P* = .62, *I*^2^ = 0%) (Figs. 13 and 14).

#### 3.4.4. Immunoregulation.

**Figure 15.**
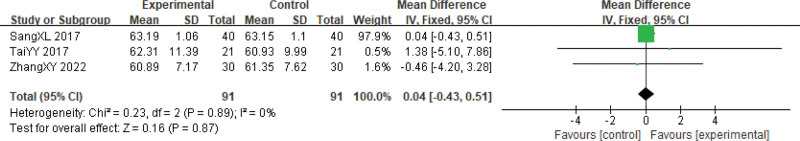
Pretreatment CD3 + levels. CI = confidence interval.

**Figure 16.**
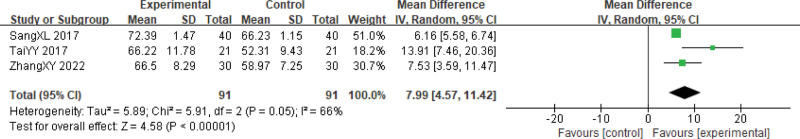
Post-treatment CD3 + levels. CI = confidence interval.

There were no significant differences in pretreatment CD3 + cell levels between the Xihuang Pills/Capsules combined with Western medicine group and the Western medicine alone group (MD = 0.04, 95% CI: –0.43 to 0.51, *P* = .87, 3 studies, 182 patients).^[24,25,33]^ Less statistical heterogeneity was observed among the studies (χ^2^ = 0.23, *P* = .89, *I*^2^ = 0%) (Fig. 15). However, CD3 + cell levels increased significantly after treatment with Xihuang Pills/Capsules in combination with Western medicine (MD = 7.99, 95% CI: 4.57–11.42, *P* < .00001, 3 studies, 182 patients). Heterogeneity resulted in χ^2^ = 5.91, *P* = .05, and *I*^2^ = 66% (Fig. 16).

**Figure 17.**
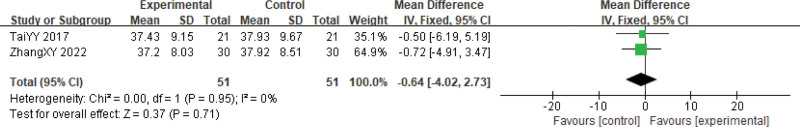
Pretreatment CD3 + CD4 + levels. CI = confidence interval.

**Figure 18.**
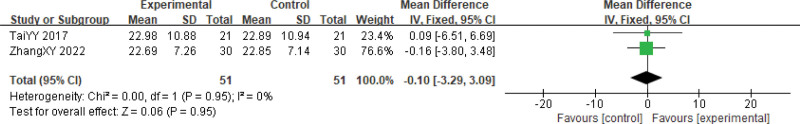
Pretreatment CD3 + CD8 + levels. CI = confidence interval.

**Figure 19.**
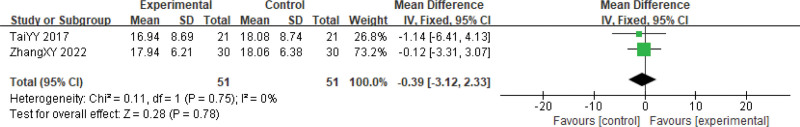
Pretreatment CD3-CD56 + levels. CI = confidence interval.

**Figure 20.**
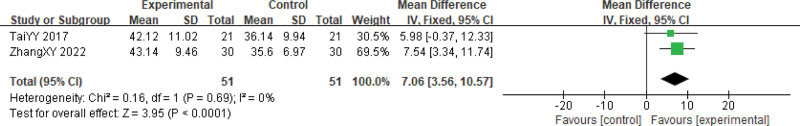
Pretreatment CD3 + CD4 + levels. CI = confidence interval.

**Figure 21.**
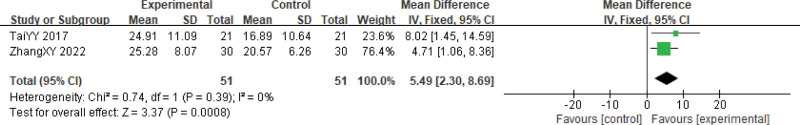
Post-treatment CD3 + CD8 + levels. CI = confidence interval.

**Figure 22.**
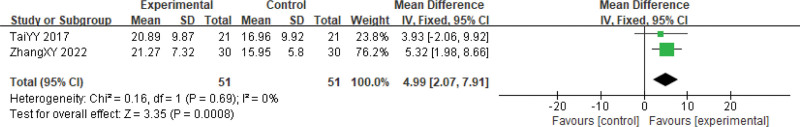
Post-treatment CD3 − CD56 + levels. CI = confidence interval.

**Figure 23.**
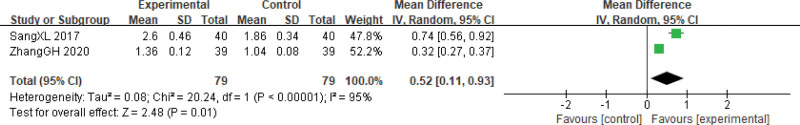
IgM levels after cervical cancer treatment. CI = confidence interval, IgM = immunoglobulin M.

Two studies reported the levels of CD3 + CD4+, CD3 + CD8+, and CD3 − CD56 + cells in both groups of 102 patients.^[24,25]^ The differences in pretreatment levels were not significant (CD3 + CD4+, MD = –0.64, 95% CI: –4.02 to 2.73, *P* = .71; CD3 + CD8+, MD = –0.10, 95% CI: –3.29 to 3.09, *P* = .95; CD3 − CD56+, MD = –0.39, 95% CI: –3.12 to 2.33, *P* = .78), and there was little statistical heterogeneity (CD3 + CD4+, χ^2^ = 0.00, *P* = .95, *I*^2^ = 0%; CD3 + CD8+, χ^2^ = 0.00, *P* = .95, *I*^2^ = 0%; CD3 − CD56+, χ^2^ = 0.11, *P* = .75, *I*^2^ = 0%) (Figs. 17–19). Additionally, the experimental group showed better post-treatment levels (CD3 + CD4+, MD = 7.06, 95% CI: 3.56–10.57, *P* < .0001; CD3 + CD8+, MD = 5.49, 95% CI: 2.30–8.69, *P* = .0008; CD3 − CD56+, MD = 4.99, 95% CI: 2.07–7.91, *P* = .0008), and there was no significant heterogeneity (CD3 + CD4+, χ^2^ = 0.16, *P* = .69, *I*^2^ = 0%; CD3 + CD8+, χ^2^ = 0.74, *P* = .39, *I*^2^ = 0%; CD3 − CD56+, χ^2^ = 0.16, *P* = .69, *I*^2^ = 0%) (Figs. 20–22). Moreover, patients treated with Xihuang Pills/Capsules in combination with Western medicine had higher immunoglobulin M levels (MD = 0.52, 95% CI: 0.11–0.93, *P* = .01, 2 studies, 158 patients).^[30,33]^ The heterogeneity test resulted in χ^2^ = 20.24, *P* < .00001, and *I*^2^ = 95% (Fig. 23). These findings indicate that combining Xihuang Pills/Capsules with Western medicine therapy might improve immune function indicators in patients with uterine cervical neoplasm.

#### 3.4.5. Prognosis of the tumor.

**Figure 24.**
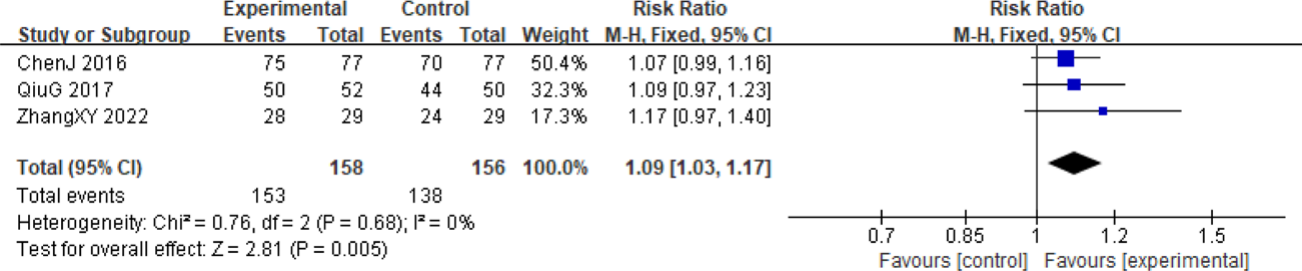
One-year survival rate. CI = confidence interval.

**Figure 25.**
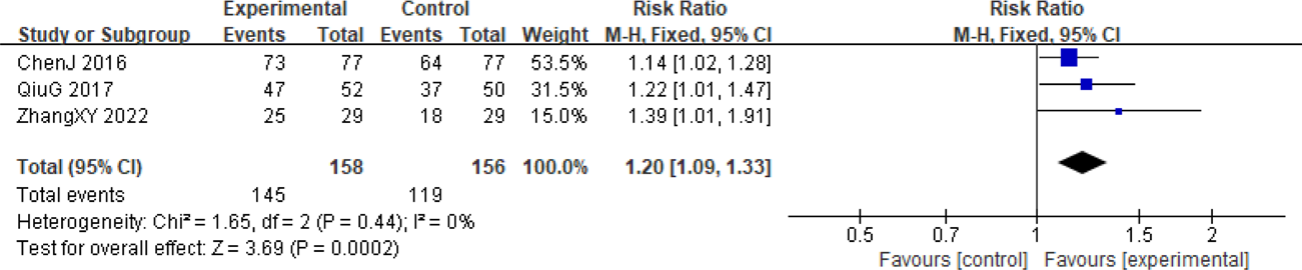
Two-year survival rate. CI = confidence interval.

Three follow-up studies^[24,27,29]^ examined the 1-year and 2-year survival rates. Two patients lost to follow-up were excluded, one from the experimental group and one from the control group.^[24]^ Among the 314 patients treated with Xihuang Pills/capsules combined with Western medicine, the 1-year and 2-year survival rates were significantly higher compared to the control group (1-year, RR = 1.09, 95% CI: 1.03–1.17, *P* = .005; 2-year, RR = 1.20, 95% CI: 1.09–1.33, *P* = .0002). Heterogeneity among studies was low (1-year, χ^2^ = 0.76, *P* = .68, *I*^2^ = 0%; 2-year, χ^2^ = 1.65, *P* = .44, *I*^2^ = 0%) (Figs. 24 and 25).

**Figure 26.**
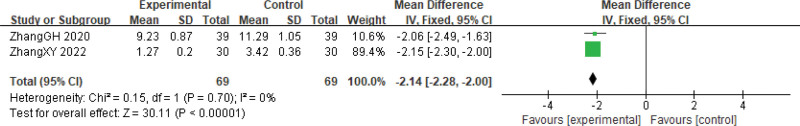
CEA after cervical cancer treatment. CEA = carcinoembryonic antigen, CI = confidence interval.

**Figure 27.**
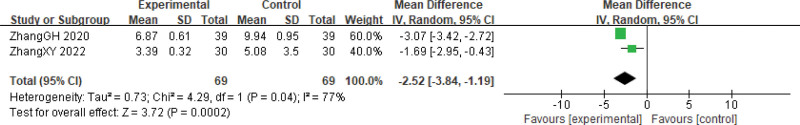
SCC-Ag after cervical cancer treatment. CI = confidence interval, SCC-Ag = squamous cell carcinoma antigen.

Two articles mentioned tumor markers, carcinoembryonic antigen (CEA) and squamous cell carcinoma antigen (SCC-Ag).^[24,30]^ In a total of 138 patients, both indicators were significantly lower in the experimental group than in the control group after receiving Xihuang Pills/Capsules treatment (CEA, MD = –2.14, 95% CI: –2.28 to 2.20, *P* < .00001; SCC-Ag, MD = –2.52, 95% CI: –3.84 to 1.91, *P* = .0002). Heterogeneity was observed between the control and experimental groups (CEA, χ^2^ = 0.15, *P* = .70, *I*^2^ = 0%; SCC-Ag, χ^2^ = 4.29, *P* = .04, *I*^2^ = 77%) (Figs. 26 and 27).

## 4. Discussion

This meta-analysis comprised 10 RCTs involving 937 participants. The findings revealed that the combination of Xihuang Pills/Capsules with Western medicine exhibited superior efficacy compared to Western medicine alone. This was evident through significant improvements in tumor response and physical functional status among patients with cervical cancer. Furthermore, the combination therapy demonstrated a substantial reduction in adverse effects, including gastrointestinal symptoms (e.g., nausea, vomiting, and diarrhea), radiation proctitis, myelosuppression, as well as inhibition of WBCs and PLTs induced by radiotherapy and/or chemotherapy. These highly encouraging results suggest that the incorporation of Xihuang Pills/Capsules with Western medicine could represent a more effective clinical approach than Western medicine alone, and might facilitate the integration of Chinese and Western medicine for uterine cervical neoplasms treatment.

The utilization of radiotherapy and chemotherapy in treating patients with cervical neoplasms significantly enhances survival rates.^[37]^ However, cancer treatment entails cytotoxicity, whether by chemotherapy or radiotherapy, leading to the destruction of tumor cells and adversely affecting normal bodily functions, resulting in various severity levels of side effects that significantly impact treatment compliance and overall quality of life.^[38]^ According to TCM theory, radiotherapy and chemotherapy may cause excessive heat toxicity, depletion of jin fluid, disharmony between yin and yang, disturbances in qi and blood circulation, and dysfunction of viscera and meridians.^[39]^

Xihuang Pills/Capsules function by stimulating blood circulation, resolving stasis, promoting orifice opening, detoxifying, softening hardness, and dispersing nodules. Some Chinese medicine experts propose that combining Xihuang Pills/Capsules with the treatment of uterine cervical neoplasms could effectively clear heat without harming yin, detoxify healthy qi, invigorate the blood without compromising qi, and exert significant anti-tumor effects.^[24]^ However, it is worth noting that most clinical efficacy studies have been based on case reports, and no evidence-based conclusions have been drawn. The objective of this meta-analysis was to provide valuable insights to guide future clinical trials of Xihuang Pills/Capsules in the treatment of cervical neoplasms.

Immune cells play a crucial role in anti-tumor therapy, with T and natural killer cell (NK) cell subsets garnering recognition from researchers worldwide. Increasing the number or enhancing the function of effector cells in patients has been found to bolster the tumor-fighting abilities of T and NK cell subsets.^[40]^ In this context, we analyzed the mean values of the T cell subset (CD3+, CD3 + CD4+, CD3 + CD8+) and NK cell subset (CD3 − CD56+) ratios using available data from both the Xihuang Pills/Capsules combined with Western medicine treatment group and the Western medicine alone group. The combination therapy group exhibited significantly higher numbers of CD3+, CD3 + CD4+, CD3 + CD8+, and CD3 − CD56 + cells. However, due to varying literature quality and small sample sizes in the included studies, it remains inconclusive whether Xihuang Pills/Capsules can alleviate tumor-induced immunosuppression by enhancing immunomodulatory function in patients. Therefore, the evidence provided above is insufficient to establish definitive conclusions. Nevertheless, the primary advantage of Xihuang Pills/Capsules as adjuvant therapy for uterine cervical neoplasms lies in its ability to enhance treatment efficacy and reduce the incidence of adverse events.

It is essential to acknowledge both the merits and limitations of this study. On the positive side, this research adhered to evidence-based medical findings, and all reviewers underwent rigorous training in high-quality meta-analysis techniques. However, all included studies were from Chinese references, which might introduce linguistic bias. Additionally, none of the trials provided detailed information on how allocation concealment or blinding was implemented, leading to a high risk of selection bias and subjective bias from investigators or participants. Moreover, the 10 included RCTs generally had small sample sizes and lacked multicenter and large studies, contributing to the overall low quality of the literature. Finally, only 3 publications reported follow-up data, which prevented the assessment of long-term efficacy and introduced the possibility of attrition.

High-grade cervical lesions resulting from HPV infection are precancerous conditions closely associated with invasive cervical cancer, necessitating conization for patients. Research data highlight that positive endocervical and/or ectocervical margins, as well as HPV persistence, are major factors affecting the risk of recurrence.^[41]^ Surgery is the primary treatment for early-stage cervical cancer, with open radical hysterectomy being the standard approach. However, studies demonstrate that minimally invasive laparoscopic surgery does not compromise survival outcomes in patients with early-stage cervical cancer.^[42]^ Further research may establish laparoscopic radical hysterectomy as an important surgical procedure for early-stage cervical cancer. For mid-to-late-stage cervical cancer, which is often accompanied by lymph node metastasis and infiltration of surrounding tissues, radical surgical treatment is often not feasible, resulting in a poor prognosis for most patients. Consequently, non-surgical options have emerged as a prominent research focus and treatment trend for middle and advanced cervical cancer.

In contrast, this study suggests that combining Xihuang Pills/Capsules with chemoradiotherapy yields favorable clinical outcomes in patients with mid-to-late-stage uterine cervical neoplasms. As integrated traditional Chinese and Western medicine continues to advance, the advantages of TCM become increasingly evident in complementing Western medicine to exert synergistic and attenuated effects, alleviate clinical symptoms in middle and advanced tumors, and enhance patients’ quality of life.^[43,44]^ To confirm the efficacy of TCM in treating cervical neoplasms, further high-quality, multicenter, large-sample, prospective, randomized, double-blind clinical trials are warranted in the future.^[3]^

## 5. Conclusion

In summary, this meta-analysis demonstrates that combining Xihuang Pills/Capsules with Western medicine for the treatment of mid-to-late-stage uterine cervical neoplasms can lead to improved patient prognosis, reduced toxic side effects, and enhanced treatment efficacy compared to Western medicine alone. Additionally, in the context of malignant tumors, Xihuang Pills/Capsules exhibit the advantage of being multi-targeted and nonresistant. However, there remains a scarcity of studies concerning the chemical composition and targets, ambiguous research on material bases, inadequate investigation into the mechanism of action, and a lack of systematic research. Moreover, the specificity of Xihuang Pills/Capsules for different types of cancer warrants further exploration. It is imperative for researchers and clinicians to assess the effectiveness of TCM in cancer treatment, elucidate the material basis and mechanism of action of Xihuang Pills/Capsules for neoplasms therapy, and furnish robust data to support their clinical application.

## Acknowledgments

The authors would like to thank all the individuals who participated in this study.

## Author contributions

**Conceptualization:** Huirong Xu, Guangyu Tian, Kejian Li.

**Data curation:** Huirong Xu, Guangyu Tian, Chunli Wu.

**Formal analysis:** Huirong Xu, Chunli Wu, Xiaowen Sun.

**Investigation:** Huirong Xu, Chunli Wu, Kejian Li.

**Methodology:** Huirong Xu, Kejian Li.

**Project administration:** Huirong Xu, Chunli Wu, Xiaowen Sun.

**Software:** Guangyu Tian, Chunli Wu, Xiaowen Sun.

**Supervision:** Huirong Xu, Xiaowen Sun, Kejian Li.

**Validation:** Guangyu Tian, Chunli Wu, Xiaowen Sun.

**Visualization:** Huirong Xu.

**Writing – original draft:** Huirong Xu, Guangyu Tian, Kejian Li.

**Writing – review & editing:** Huirong Xu, Guangyu Tian, Kejian Li.
